# Hemodynamic Monitoring Today

**DOI:** 10.1155/2011/535912

**Published:** 2011-09-25

**Authors:** Maurizio Cecconi, Jamal A. Alhashemi, Maxime Cannesson, Christoph K. Hofer

**Affiliations:** ^1^Department of General Intensive Care, St George's Hospital and Medical School, London SW17 0QT, UK; ^2^Department of Anesthesia and Critical Care, King Abdulaziz University Hospital, 21589 Jeddah, Saudi Arabia; ^3^Department of Anesthesiology and Perioperative Care, School of Medicine, University of California, Irvine, Orange CA 92868, USA; ^4^Institute of Anesthesiology and Intensive Care Medicine, Triemli City Hospital, 8063 Zurich, Switzerland

Hemodynamic monitoring has been part of the routine management of intensive care patients and high risk surgical patients since the advent of the pulmonary artery catheter (PAC) more than thirty years ago. The growing availability of new less invasive devices over the past decades has now made it possible to monitor cardiac output (CO) more often in the operating room, as well as in new clinical settings such as the emergency department. 

In this special issue papers have been arranged that discuss different aspects of hemodynamic monitoring, from technical issues to new clinical applications.

The first paper titled “*Perioperative intravascular fluid assessment and monitoring: a narrative review of established and emerging techniques*” is a review on perioperative intravascular fluid assessment and monitoring. How cardiovascular physiology can be monitored is explained, and different technologies are presented.

The second paper titled *“Cardiac output assessed by invasive and minimally invasive techniques”* is a review on all available technologies to monitor CO. The authors start from the PAC and carry on to present the more recent less invasive devices. The review covers technical aspects as well as clinical validation and use.

The third paper titled “*The effect of a hyperdynamic circulation on tissue Doppler values: a simulation in young adults during exercise*” presents research done on healthy individuals undergoing strenuous exercise, using left ventricular tissue Doppler velocity (TDI), giving us new data on the use of TDI in hyperdynamic circulation.

The fourth paper titled “*The effect of airway pressure release ventilation on pulmonary catheter readings: specifically pulmonary capillary wedge pressure in a swine model*” investigates the effect of the airway pressure release ventilation (APRV) on the PAC readings in an animal model. This papers gives new insights into the heart-lung interaction when using this new mode of ventilation. 

In the fifth paper titled “*Clinical applications of heart rate variability in the triage and assessment of traumatically injured patients*” heart rate variability (HRV) is explored. HRV represents a way of looking at the function of autonomic nervous system during stress conditions and has been studied in different areas as a predictor of morbidity and mortality. In this paper the authors focus on the use of HRV in trauma patients.

The sixth paper titled “*Recommendations for haemodynamic and neurological monitoring in repair of acute type A aortic dissection*” gives a perspective on how hemodynamic monitoring should be used in conjunction with other clinical strategies. In this review the authors describe how hemodynamic monitoring can be combined with neurological monitoring in order to optimize the circulation guaranteeing adequate cerebral blood flow. 

The seventh paper titled “*Assessing the left ventricular systolic function at the bedside: the role of transpulmonary thermodilution-derived indices*” is a review on the use of transpulmonary thermodilution to measure CO and other derived variables. The authors focus on how these variables can be used to assess the left ventricular function in different clinical situations.

*Maurizio Cecconi* *Jamal A. Alhashemi* *Maxime Cannesson* *Christoph K. Hofer*

